# Genome-Wide Identification of the Chalcone Synthase Gene Family in *Phoebe zhennan* and Its Association with Golden-Thread Wood Color Formation and Drought Response

**DOI:** 10.3390/genes17091027

**Published:** 2026-08-28

**Authors:** Kui Yan, Shuai He, Jian Peng, Jianghong Qian, Yunjie Gu, Hongying Guo, Suyuan Zhang, Yulin Wei, Ruiqi Wang, Yu Zhou, Hanbo Yang

**Affiliations:** 1 Ecological Conservation, Restoration and Resource Utilization in Forest and Wetland Key Laboratory of Sichuan Province, Sichuan Academy of Forestry, Chengdu 610081, China; 2 Ecology and Conservation in the Upper Reaches of the Yangtze River Key Laboratory of Sichuan Province, Sichuan Mt. Emei Forest Ecosystem National Observation and Research Station, College of Forestry, Sichuan Agricultural University, Chengdu 611130, China

**Keywords:** *P. zhennan*, chalcone synthase, genome-wide analysis, golden-thread wood color, drought response

## Abstract

Chalcone synthase (CHS) is a key enzyme in flavonoid biosynthesis, and its activity strongly influences the efficiency of flavonoid production in plants. By controlling the biosynthesis of secondary metabolites, CHS contributes to plant adaptation to diverse environmental stresses. Here, we identified CHS members across the *Phoebe zhennan* genome and systematically examined their gene structures, phylogenetic relationships, and potential roles in golden-thread wood color formation and drought response. Genome-wide screening resolved 11 putatively functional PzCHSs, which were unevenly distributed across six chromosomes. Physicochemical analyses indicated that most PzCHS proteins were hydrophilic. Phylogenetic reconstruction divided the PzCHS family into three groups, whose members generally shared similar gene structures. One tandem duplication event and five segmental duplication events were detected. Synteny analysis revealed extensive collinearity between the CHS families of *P. zhennan*, *Phoebe bournei* and *Cinnamomum camphora*, consistent with their close evolutionary relationships. Promoter regions of PzCHS genes were enriched for phytohormone-responsive and growth-related cis-elements, suggesting roles in development and hormonal regulation. Transcriptome profiling showed that PzCHS6 was upregulated in the transition zone of wood and under drought stress, suggesting that this gene may be associated with both golden-thread wood color formation and drought response. These results provide a framework for functional dissection of the CHS members and offer candidate genes for golden-thread wood color improvement and drought response breeding.

## 1. Introduction

Chalcone synthase (CHS) is a key catalytic enzyme in the phenylpropanoid pathway and initiates flavonoid backbone formation through substrate-specific reactions. Its primary products undergo chalcone isomerase (CHI)-mediated structural rearrangement, enabling the biosynthesis of diverse flavonoids [1]. CHS catalysis begins when coumaroyl-CoA binds to a cysteine residue in the CHS active center through a thioester bond. Subsequent condensation and decarboxylation of malonyl-CoA generate a 15-carbon C6-C3-C6 intermediate. Cyclization of the polyketide chain forms an aromatic ring, followed by isomerization into flavanones and downstream production of flavones and flavonols [2,3]. Previous studies have also shown that CHS members regulate plant pigment synthesis through flavonoid biosynthesis and participate in abiotic stress responses. In citrus, three functional CitCHS members show distinct expression patterns and contribute differently to flavonoid production [1]. In maize, *ZmCHS25* knockout reduced flavonoid biosynthesis, disrupted phenylpropanoid metabolism and impaired osmotic regulation under salt stress, linking CHS activity directly to stress-associated metabolic control [4]. With advances in whole-genome sequencing and assembly, genome-wide identification of gene families has become essential for understanding family composition, evolution and function [5]. CHS members have been identified and analyzed in many plants, including *Arabidopsis thaliana* [6], *Juglans regia* [7], and *Cucumis sativus* [8].

Wood formation begins with the proliferation of cambial derivatives and ends with mature xylem fibers and vessels bearing lignified secondary cell walls, reflecting coordinated physiological, biochemical and molecular processes [9,10]. Among the traits that emerge from this process, wood color is a fundamental trait and a major determinant of timber quality and market value [11,12]. Wood color arises from chromophores dispersed or deposited in xylem cells. Pigment molecules in lignin and extractives, including pigments, tannins and resins, absorb ultraviolet and visible radiation from sunlight and thereby generate visible color [13,14]. Among them, flavonoids are among the principal plant pigments and are one of the most important pigment classes in wood [13,15]. For instance, the accumulation and release of flavonoids such as luteolin, gnaphaliin, dalbergin and dalbergiphenol in the xylem cells of *Cunninghamia lanceolata*, *Dalbergia odorifera*, and *Dalbergia latifolia* are major contributors to red, dark-brown, dark-purple and yellow wood colors [11,12,13,16].

Global warming is intensifying, and extreme drought events are becoming more frequent, posing serious threats to agricultural production and ecosystems [17]. Research on plant drought response is therefore increasingly urgent. As terminal products of the phenylpropanoid pathway, flavonoids contribute to plant drought responses by coordinating reactive oxygen species (ROS) scavenging and stomatal movement, thereby building a multilayered defense network [18]. Under drought stress, excessive ROS accumulation causes cellular damage. Flavonoids can directly scavenge superoxide anions, hydrogen peroxide and other radicals, while activating antioxidant enzyme systems to alleviate oxidative stress [18]. Flavonoids can also help maintain cellular water potential by reducing transpiration and activate drought-related transcription factors through stress-responsive elements [19,20]. Clarifying flavonoid biosynthesis is therefore important for wood color improvement and drought-resistance breeding in forest trees.

*Phoebe zhennan*, a tall evergreen tree in Lauraceae, is a valuable native species endemic to China [21,22,23]. Its timber is known as “golden-thread nanmu” because of its golden, silky luster and is widely regarded as one of the finest sources of this prized wood [24,25]. Our previous work showed that flavonoids are key pigments underlying the characteristic of golden-thread wood color in *P. zhennan* [26,27,28]. As a key enzyme in flavonoid biosynthesis, CHS may be involved in golden-thread wood color formation and abiotic stress responses of *P. zhennan*. In this study, we identified CHS members genome-wide in *P. zhennan*, characterized their phylogenetic relationships and gene structures, and analyzed transcript profiles across golden-thread wood formation and drought stress treatments. This integrated analysis provides candidate genes and a theoretical basis for improving wood color and drought response in *P. zhennan*.

## 2. Materials and Methods

### 2.1. Identification of CHS Members

Hidden Markov model (HMM) profiles for the chalcone synthase (CHS) gene family (IPR001099 and IPR012328) were downloaded from Pfam. These profiles were used for hmmsearch against the *P. zhennan* genome (https://doi.org/10.6084/m9.figshare.28218275) [26] with a threshold of 10.0 and the cut-off value was set to 0.01, yielding two preliminary sequence datasets. After merging and redundancy removal, the first dataset of candidate PzCHS members was obtained. The CHS protein sequences of *A. thaliana* (https://ftp.ncbi.nlm.nih.gov/genomes/all/GCA/000/001/735/GCA_000001735.2_TAIR10.1/, accessed on 26 August 2026) with confirmed annotations were then downloaded and used as queries for BLASTP searches against the *P. zhennan* protein database, generating a second candidate dataset. The two datasets were merged, deduplicated and checked for conserved domains using Pfam, SMART and NCBI-CDD to obtain the final set of Pz CHS members. The CHS members in *Populus trichocarpa* (version 4.1) (https://ftp.ncbi.nlm.nih.gov/genomes/all/GCA/000/002/775/GCA_000002775.4_P.trichocarpa_v4.1/, accessed on 26 August 2026) [29], *Eucalyptus grandis* (version 2.0) (https://phytozome-next.jgi.doe.gov/info/Egrandis_v2_0, accessed on 26 August 2026) [30], *Phoebe bournei* (v1.0) (https://db.cngb.org/search/assembly/CNA0029376/, accessed on 26 August 2026) [31], and *Cinnamomum camphora* (https://ngdc.cncb.ac.cn/search/?dbId=gwh&q=PRJCA005135&page=1, accessed on 26 August 2026) [32] were identified based their genome datasets using the same procedure.

### 2.2. Physicochemical Properties and Subcellular Localization Prediction

The ExPASy-ProtParam online platform (http://web.expasy.org/protparam/, accessed on 26 August 2026) was used to evaluate physicochemical properties, including polypeptide length (aa), molecular weight (MW), theoretical isoelectric point (pI), grand average of hydropathy (GRAVY), aliphatic index (AI) and instability index (II) [33]. Subcellular localization was predicted with WoLF PSORT [34]. Three-dimensional protein structures of family members were modeled using homology modeling on the Swiss-Model online platform [35].

### 2.3. Phylogenetic Analysis

Multiple sequence alignment of CHS members was performed with ClustalW [36]. The resulting alignment was used to construct a maximum-likelihood phylogenetic tree with IQ-TREE [37]. The best fitting amino acid substitution model was selected and branch support was evaluated with 1000 bootstrap replicates. No additional alignment trimming was applied. The phylogenetic tree was visualized on the Evolview platform [38].

### 2.4. Chromosomal Localization and Synteny Analysis

Chromosomal localization of PzCHS members was analyzed and visualized with TBtools-II [39]. Duplication events were identified with the Multiple Collinearity Scan toolkit (MCScanX) [40] and visualized with TBtools. Syntenic relationships between PzCHSs and CHS members from *P. trichocarpa*, *E. grandis*, *C. camphora*, *P. bournei*, and *A. thaliana* were analyzed using MCScanX and visualized with TBtools. The nonsynonymous substitution rate (Ka), synonymous substitution rate (Ks) and Ka/Ks ratio for each duplicated CHS pair were calculated using KaKs_Calculator 2.0 [41].

### 2.5. Conserved Motif and Gene Structure

Protein sequences of PzCHS members were analyzed for conserved motifs using MEME [42]. Parameters were set to any number of repetitions, a maximum of 20 motifs and an optimal motif width of 6–100 amino acid residues. Predicted conserved motifs were visualized using WEBLOGO [43] and TBtools. Exon–intron structures were analyzed and visualized using GSDS 2.0 [44].

### 2.6. Analysis of Promoter Cis-Acting Elements

Promoter sequences 2000 bp upstream of the start codon of each PzCHS member were extracted. Cis-acting elements were predicted using the PlantCARE database [45] and visualized with TBtools.

### 2.7. Transcript Expression Patterns Across Different Stem Xylem Regions

To investigate the potential roles of *PzCHS* members in golden-thread wood color formation, we analyzed their transcript expression patterns across different stem regions based our previously generated transcriptome data. The transcriptome data from different stem regions were taken from PRJNA898439 [28] and CRA022193 [26]. All samples were collected from 80-year-old *P. zhennan* trees growing at Chengdu Du Fu Thatched Cottage, Sichuan Province, China (30°39′37.00″ N, 104°10′41.69″ E). For PRJNA898439, the outer sapwood (SW1), inner sapwood (SW2), and transition zone (TZ) were collected in 2021 from six 80-year-old *P. zhennan* trees (six biological replicates). For CRA022193, the phloem (VC), secondary xylem (SX), and primary xylem (PX) were collected at three time points (February, April, and June 2024) from five 80-year-old *P. zhennan* trees (five biological replicates). Because these datasets were generated from different sampling years, tissue types and experimental designs, expression patterns were interpreted within each dataset rather than used for direct quantitative comparison across datasets. The expression level of each PzCHS member was represented by FPKM values calculated using HISAT2 [46] and StringTie [47]. For heatmap visualization, FPKM values were further standardized by Z-score normalization to show relative expression patterns within each dataset. Pairwise comparisons among different stem xylem regions were performed using replicate-level FPKM values. *p* values were calculated using Welch’s t-test, and multiple testing correction was performed using the Benjamini–Hochberg method to obtain false discovery rate (FDR) values. Significantly differentially expressed genes (DEGs) were identified using thresholds of an FDR < 0.05 and a |log2FC| ≥ 1.

### 2.8. Transcript Expression Patterns Under Drought Stress

To investigate the potential roles of *PzCHS* members in drought stress responses, we analyzed their transcript expression patterns under drought stress based on previously generated transcriptome data. The transcriptome data for drought stress were taken from the NCBI SRA database (PRJNA778346) [48]. For drought stress treatment, two-year-old *P. zhennan* seedlings with similar growth status were grown in plastic pots containing nutrient soil under controlled grown chamber conditions and then subjected to natural drought stress by water withholding. Leave samples were collected before drought treatment (day 0) and after 16 days of drought treatment, with three biological replicates [48]. FPKM values under drought stress were calculated using the same HISAT2- and StringTie-based pipeline described above, and the resulting FPKM values were used for heatmap visualization after Z-score normalization. The DEGs were identified using the same criteria as described in Section 2.7.

## 3. Results

### 3.1. Identification and Sequence Analysis of CHS Members

Eleven non-redundant PzCHS proteins were identified in the *P. zhennan* genome, which was broadly comparable to that in *A. thaliana* (10), *P. trichocarpa* (19), *E. grandis* (13), *C. camphora* (10) and *P. bournei* (13) (Table 1 and Table S1). According to chromosomal position, these CHS members were named PzCHS1-PzCHS11 (Table S1). Across the six plants examined, acidic proteins (pI < 7) accounted for 59.2% of members, whereas basic proteins (pI > 7) accounted for 40.8%. Among them, the PzCHS members included six acidic and five basic proteins. Stable proteins (II < 40) and unstable proteins (II > 40) accounted for 51.3% and 48.7%, respectively, with five stable and six unstable proteins in *P. zhennan*. Hydrophilic and hydrophobic proteins accounted for 88.2% and 11.8%, respectively; the PzCHS members contained eight hydrophilic and three hydrophobic proteins. Among PzCHS proteins, amino acid length, molecular weight and pI ranged from 384 (PzCHS9) to 532 (PzCHS5), 42.52 kDa (PzCHS9) to 59.36 kDa (PzCHS5), and 5.81 (PzCHS7) to 9.46 (PzCHS5), respectively. Subcellular localization predictions indicated that most CHS proteins were localized to the cytoplasm, plasma membrane, or chloroplast, whereas fewer members were predicted to localize to the endoplasmic reticulum, mitochondria or nucleus. Three-dimensional structural prediction showed that members with similar physicochemical properties had similar predicted structures, and the main conformations of each PzCHS protein consisted of alpha-helices and beta-sheets (Figure S1).

### 3.2. Multiple Sequence Alignment and Phylogenetic Analysis

**Figure 1:**
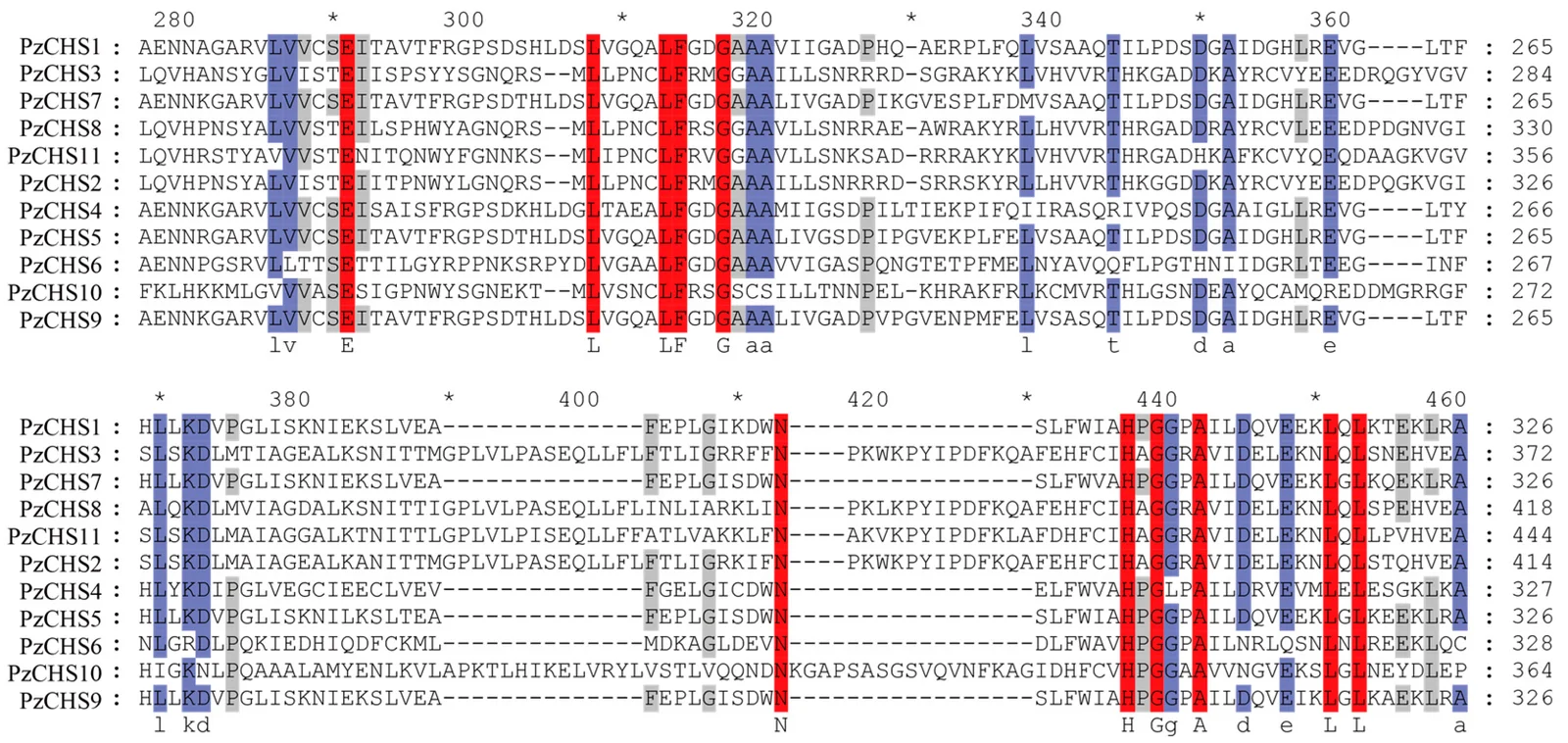
Partial domain amino acid sequences of PzCHS proteins.

**Figure 2:**
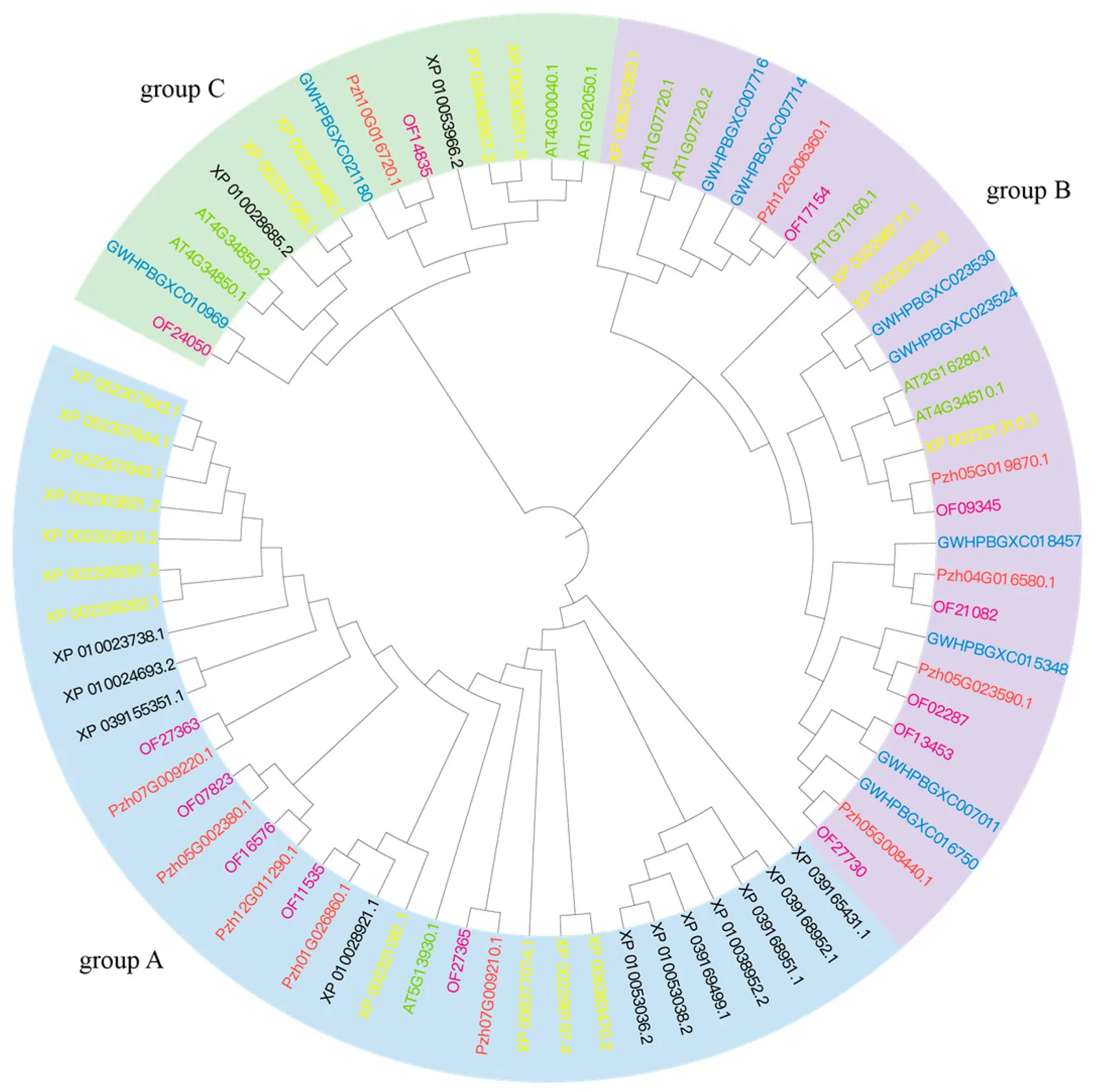
Phylogenetic analysis of CHS members from six plants. CHS proteins from *Arabidopsis thaliana*, *Eucalyptus grandis*, *Cinnamomum camphora*, *Populus trichocarpa*, *Phoebe bournei*, and *Phoebe zhennan* are shown in green, black, blue, yellow, pink and red fonts, respectively.

Multiple sequence alignment performed using ClustalW revealed an overall amino acid sequence similarity of 28.17% among PzCHS proteins (Figure 1). PzCHS1, PzCHS3, PzCHS7, PzCHS8 and PzCHS11 showed high sequence similarity to one another, as did PzCHS2, PzCHS4, PzCHS5, PzCHS6 and PzCHS10. By contrast, PzCHS9 were shown to be relatively divergent from the other family members. Phylogenetic analysis grouped the CHS members into three groups (Figure 2). Group A contained PzCHS1, PzCHS3, and PzCHS7, PzCHS8, and PzCHS11, whereas group B included PzCHS2, PzCHS4, and PzCHS5, PzCHS6, and PzCHS10. Group C contained only PzCHS9. The PzCHS proteins were more closely related to homologues from *P. bournei* and *C. camphora*, consistent with the placement of these species within Lauraceae.

### 3.3. Gene Structure and Conserved Motifs

**Figure 3:**
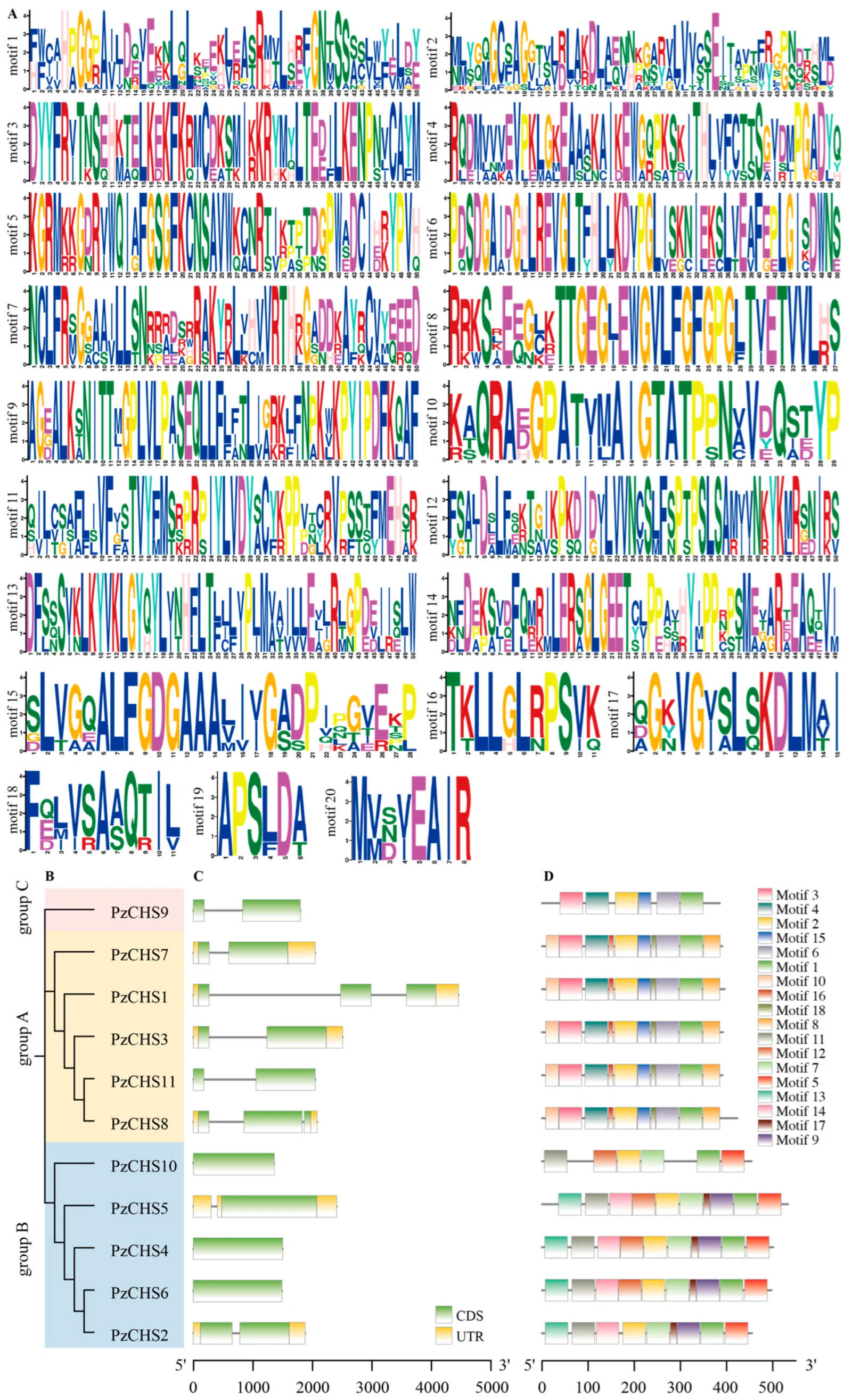
Gene structures and conserved motifs of PzCHS members. (**A**) Conserved motifs of PzCHS proteins. The height of each amino acid letter reflects its frequency in the conserved motif sequence, the *x*-axis indicates amino acid position, and the *y*-axis indicates conservation. (**B**) Phylogenetic tree constructed from full-length PzCHS sequences. (**C**) Structural features of PzCHS genes. (**D**) Distribution of conserved domains in PzCHS members.

Conserved motifs in PzCHS proteins were identified with MEME, yielding 20 motifs named motif 1 to motif 20 (Figure 3A). Motif lengths ranged from 6 to 50 amino acid residues. The 11 PzCHS members were divided into three groups (Figure 3B). The number of exons ranged from one (*PzCHS4* and *PzCHS6*) to three (*PzCHS1* and *PzCHS8*). Five members (PzCHS4, PzCHS6, PzCHS9, PzCHS10, and PzCHS11) lacked 5′ UTR and 3′ UTR regions (Figure 3C). Members within the same group showed similar structure features. For example, three PzCHSs in group A contained UTR regions, whereas the PzCHSs in group B generally contained one or two coding sequences (CDS). PzCHSs from the same group usually had similar motif compositions (Figure 3D). Motif 1 and motif 2 occurred in every family member. Motif 5 and motif 11 were present only in group B. Motif 8, motif 10, motif 16 and motif 18 were common in group A, and the presence or absence of these four motifs was the main feature distinguishing group A from group C. The PzCHSs from both group A and group B contained 10 conserved motifs, indicating relatively rich motif repertoires. The conserved motif types in group A were highly consistent, suggesting strong conservation of this group during evolution.

### 3.4. Chromosomal Distribution, Synteny Analysis, and Gene Duplication Events

**Figure 4:**
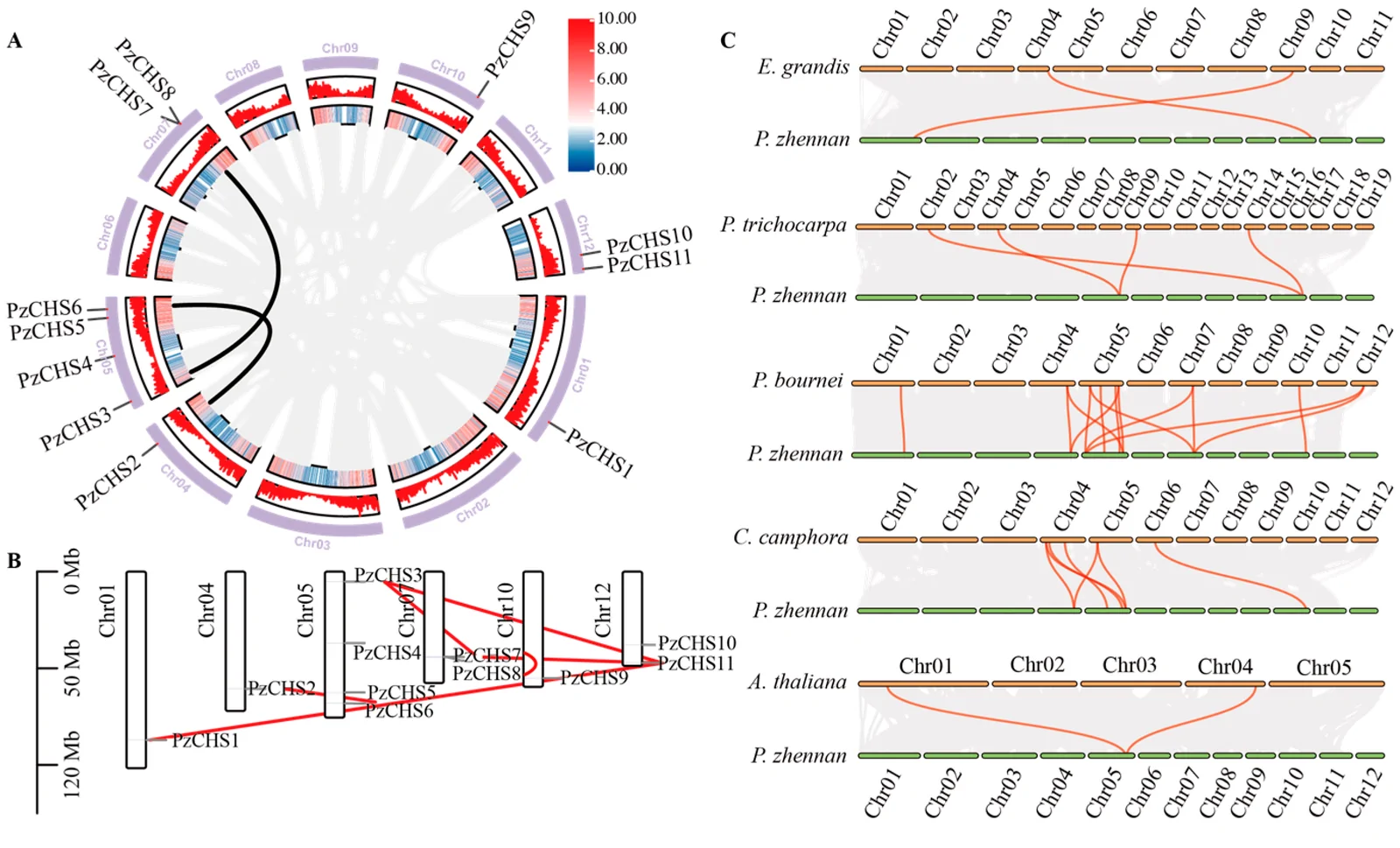
Chromosomal distribution, synteny and duplication events of PzCHSs. (**A**) Chromosomal distribution and intra-family synteny of PzCHSs. From the center to the outside, the circular plot shows the gene density heat map, gene density line plot and chromosomes. Gray lines indicate homologous genomic blocks, and black lines highlight syntenic relationships among duplicated PzCHS members. (**B**) Chromosomal distribution and duplication events of PzCHSs. Red arcs indicate tandem duplications, and red lines indicate segmental duplications between genes. (**C**) Cross-species synteny analysis of CHS members between *P. zhennan* and five representative plants. Red lines indicate homologous CHS pairs; background gray lines represent syntenic blocks between *P. zhennan* and other plant genomes.

**Figure 5:**
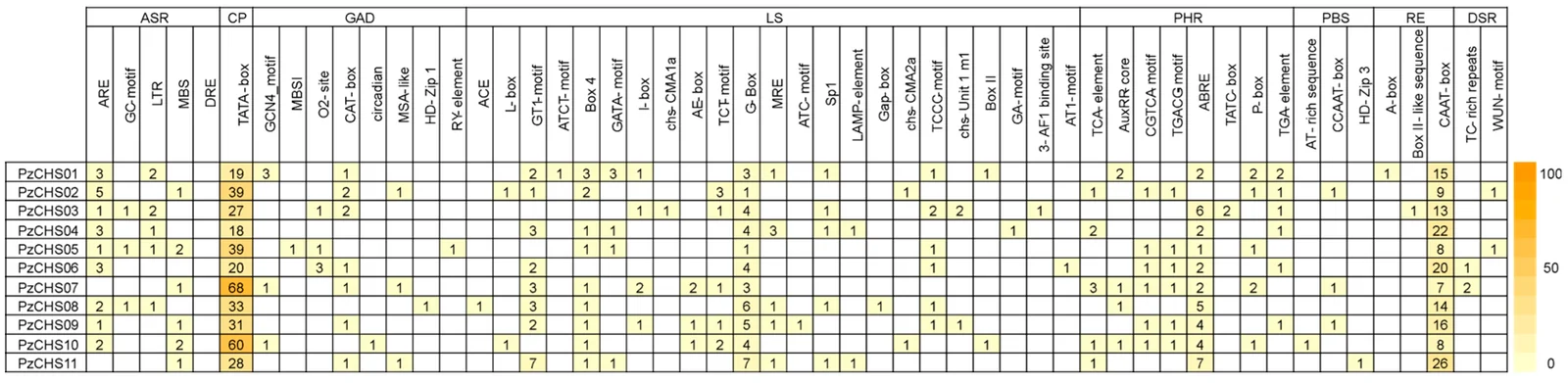
Cis-acting elements in promoter sequences of PzCHS members. ASR, abiotic stress response element; LS, light response element; PHR, phytohormone response element; GAD, growth and development element; PBS, protein-binding site; RE, regulatory element; DSR, defense and stress response element; CP, core promoter element.

The *PzCHSs* were unevenly distributed across six chromosomes, with one to four *PzCHSs* per chromosome (Figure 4A). Correlation analysis showed no relationship between chromosome length and the number of *PzCHSs* (*r* = 0.204, *p* = 0.526). A tandem duplication event was detected between *PzCHS7* and *PzCHS8* on Chr07 (Figure 4B). Five segmental duplication events were also detected: *PzCHS2* and *PzCHS6*, *PzCHS3* and *PzCHS7*, *PzCHS1* and *PzCHS11*, *PzCHS3* and *PzCHS11*, and *PzCHS7* and *PzCHS11*. Synteny analysis revealed collinearity between *PzCHS2* and *PzCHS6*, *PzCHS3* and *PzCHS7*, and *PzCHS3* and *PzCHS8* (Figure 5). *PzCHSs* showed extensive synteny with those of *P. bournei* and *C. camphora*, consistent with their close phylogenetic relationships (Figure 4C). Analysis of nonsynonymous (Ka) and synonymous (Ks) substitution rates showed that Ka/Ks ratios for duplicated *PzCHS* pairs ranged from 0.060 to 0.523, all below 1.000 (Table 2). This pattern indicates that these gene pairs have experienced purifying selection during evolution.

### 3.5. Promoter Cis-Acting Elements Analysis

Promoter sequences of *PzCHSs* were enriched for multiple light-responsive elements, core promoter elements (TATA-boxes), and functional elements involved in growth, development and phytohormone responses (Figure 5). Of which, growth and development-related elements included modules associated with circadian regulation, meristem-specific expression (CAT-box), cell cycle regulation (MSA-like), and regulation of flavonoid biosynthetic genes (MBSI). Phytohormone-responsive elements included methyl jasmonate-responsive elements (CGTCA-motif and TGACG-motif), gibberellin-responsive elements (TATC-box and P-box), ABA-responsive elements (ABRE), salicylic acid-responsive elements (TCA-element), and auxin-responsive elements (TGA-element and AuxRR-core). Defense and stress-responsive elements, including TC-rich repeats and WUN-motif, were also detected. All promoters of *PzCHSs* contained at least one light-responsive element. G-Box was the most abundant element (14.1%), followed by Box 4 (11.5%), GT1-motif (10.3%) and TCCC-motif (7.7%). These results indicate that *PzCHS* promoters contain diverse predicted cis-elements potentially associated with light, developmental, hormonal and stress-related regulation.

### 3.6. Transcript Profiles of PzCHSs Across Stem Regions and Under Drought Stress

**Figure 6:**
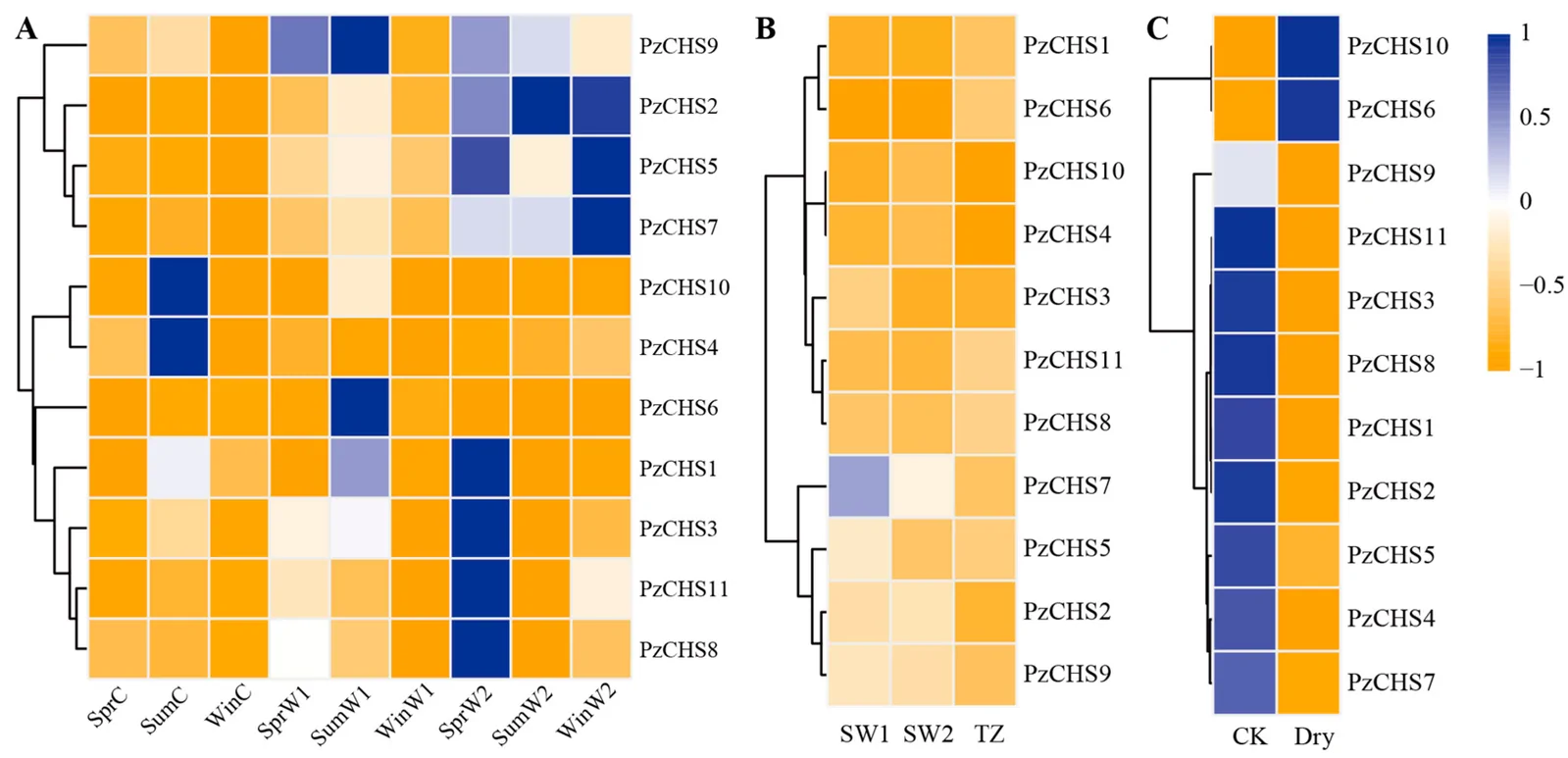
Expression profiling of *PzCHSs* in different stem regions and under drought stress. (**A**) Expression profiling of *PzCHS* members in different stem regions at different development stages. SprC, SprW2, and SprW1 represent the phloem, secondary xylem, and primary xylem, respectively, in the February group. SumC, SumW2, and SumW1 represent the phloem, secondary xylem, and primary xylem, respectively, in the April group. WinC, WinW2, and WinW1 represent the phloem, secondary xylem, and primary xylem, respectively, in the June group. (**B**) Expression profiling of *PzCHS* members in different xylem regions. SW1, outer sapwood. SW2, inner sapwood. TZ, transition zone. (**C**) Expression profiling of *PzCHS* members under drought stress. CK, the control group, refers to seedlings at day 0 without drought stress. Dry refers to seedlings subjected to drought stress for 16 days.

To explore the molecular functions of *PzCHS* members associated with golden-thread wood color formation and drought stress responses, we determined the transcript profiles of *PzCHS* members across stem regions and under drought stress using previously published transcriptome data (Figure 6). The results showed that distinct expression divergence among *PzCHS* members across xylem developmental tissues and stages (Figure 6A, Table S2). Several PzCHS genes showed elevated expression in primary and secondary xylem, with *PzCHS1*, *PzCHS3*, *PzCHS8*, and *PzCHS11* exhibiting relatively high expression in xylem tissues at the stage of April, and *PzCHS2*, *PzCHS5*, and *PzCHS7* being strongly upregulated in primary xylem. *PzCHS* members showed distinct spatial expression differences among different xylem regions (Figure 6B, Table S3). Some *PzCHS* genes exhibited relatively high expression in sapwood (SW1 and SW2) or the transition zone (TZ). Among them, *PzCHS7* was highly expressed in the SW region, while *PzCHS1*, *PzCHS6*, *PzCHS8*, and *PzCHS11* showed relatively high expression in TZ. Integrated analysis of two transcriptome datasets from different xylem tissues showed that *PzCHS2*, *PzCHS5*, *PzCHS7*, and *PzCHS9* were consistently upregulated in sapwood. Given that CHS encodes a key enzyme in the flavonoid biosynthetic pathway, the elevated expression of these genes may be associated with the biosynthesis and accumulation of flavonoids in sapwood, which may subsequently be deposited in the heartwood during the transition from sapwood to heartwood, thereby contributing to golden-thread wood color formation. Relative to the day 0 pre-treatment baseline, *P. zhennan* seedlings after 16 days of drought treatment showed distinct drought-associated expression patterns among *PzCHS* members (Figure 6C, Table S4). *PzCHS6* and *PzCHS10* were significantly upregulated relative to the day 0 baseline, whereas the other *PzCHS* members showed an overall downregulated expression pattern. These results suggest that the induced expression of *PzCHS6* and *PzCHS10* may be associated with the accumulation of flavonoids under drought stress, thereby contributing to drought response in seedlings.

## 4. Discussion

Chalcone synthase (CHS), a key rate-limiting enzyme in flavonoid biosynthesis, has a central regulatory role in plant pigment formation [1,8]. The number of CHS members varies among plant species, generally ranging from approximately 7 to 35 [49,50]. In this study, 11 CHS members were identified genome-wide in *P. zhennan*, a number comparable to those in *A. thaliana* (10), *P. trichocarpa* (19), *E. grandis* (13), *C. camphora* (10) and *P. bournei* (13), suggesting that the CHS family in *P. zhennan* is relatively conserved and has not undergone large-scale gene duplication or loss. Most PzCHS proteins were predicted to function in the cytoplasm and plasma membrane, indicating that their regulatory activities may occur mainly in these cellular compartments. Phylogenetic analysis is a core tool for resolving gene family evolutionary history, functional divergence and species adaptation [51]. It can trace gene origins and evolutionary trajectories and provide a theoretical basis for functional annotation and genetic improvement [52,53]. The PzCHS members were divided into three groups, and members within the same group had similar gene structures. Exon number can influence protein diversity and functional complexity [54]. *PzCHSs* contained one to three exons, with structural conservation within groups. Tandem duplication is often caused by replication slippage or transposons and can generate adjacent duplicated genes with redundant or diverged functions. Segmental duplication is commonly triggered by non-homologous recombination or remnants of genome duplication, promoting new gene origins or chromosomal structural reshaping [55,56]. Tandem and segmental duplications are important mechanisms contributing to gene family expansion and genome evolution [57]. The coexistence of tandem and segmental duplication events in the PzCHS family suggests that both mechanisms may have contributed to its evolutionary history, although their effects on functional divergence and environmental adaption remain to be experimentally determined [51]. *PzCHSs* showed stronger synteny with *P. bournei*, a congeneric species, and *C. camphora*, a species in the same family, consistent with their close relationships. Ka and Ks analyses are widely used to infer evolutionary pressure, functional conservation and adaptation. Their ratios and site-level patterns provide key evidence for natural selection in evolutionary biology, functional genomics and related fields [58,59]. Here, all duplicated PzCHS pairs had Ka/Ks ratios below 1.0, indicating purifying selection and strong evolutionary constraint.

Analysis of cis-acting elements can provide preliminary clues to potential transcriptional regulation, spatiotemporal expression patterns and adaptation response, and may help prioritize candidate genes for future functional studies [60,61]. The promoters of *PzCHS* genes contained multiple predicted light-responsive, growth and development-related, and phytohormone-responsive elements. These patterns suggest that *PzCHS* genes may be transcriptionally responsive to developmental, environmental and hormonal signaling. Flavonoids are important pigment compounds involved in wood color formation, and CHS, as a key gene in the flavonoid biosynthetic pathway, has transcriptional expression levels closely associated with wood coloration [16,26,62]. In *P. zhennan* wood formation, *PzCHS1*, *PzCHS6*, *PzCHS8*, and *PzCHS11* were relatively upregulated in the transition zone, suggesting that these genes may be associated with flavonoid biosynthesis during the transition from sapwood to heartwood and potentially contribute to flavonoid accumulation in heartwood, thereby contributing to golden-thread heartwood color formation. In contrast, *PzCHS2*, *PzCHS5*, *PzCHS7*, and *PzCHS9* were consistently upregulated in sapwood, indicating that they may enhance flavonoid biosynthesis and accumulation in sapwood, with these compounds subsequently being deposited in both sapwood and heartwood regions and participating in golden-thread wood coloration. In addition to its role in flavonoid-mediated pigmentation, CHS is also widely involved in plant responses to abiotic stresses by regulating the biosynthesis of stress-related flavonoid compounds. In *Medicago truncatula*, drought stress significantly induced the expression of *MtCHS1*, *MtCHS9*, and *MtCHS10*, accompanied by increased total flavonoid accumulation and antioxidant capacity, suggesting a close link between CHS induction, flavonoid biosynthesis, and antioxidant defense [63]. Consistently, the drought-induced upregulation of *PzCHS6* and *PzCHS10* in *P. zhennan* seedlings suggests that specific *PzCHS* members may play important roles in flavonoid-mediated drought response. Notably, *PzCHS6* was upregulated both in transition zone of wood and under drought stress, suggesting that this gene may be associated with both golden-thread wood formation and drought stress response in *P. zhennan*. However, the functional involvement of *PzCHS6* in golden-thread wood color formation and drought response remains associative rather than causal. Future studies of gene function analysis, such as in vitro enzymatic assays and transgenic experiments, will be required to clarify the precise biological roles of candidate *PzCHS* genes in *P. zhennan*.

## Figures and Tables

**Table 1:** Physicochemical properties and subcellular localization prediction of CHS members.

Species	Numbers	PL (aa)	MW (kDa)	pI	GRAVY	AI	II	Subcellular Localization					
								E.R.	chlo	plas	cyto	mito	nucl
*Phoebe zhennan*	11	384–532	42.52–59.36	5.81–9.46	−0.068	88.89–105. 1	35.34–46.87	1	2	4	4	0	0
*Arabidopsis thaliana*	10	319–521	34.92–54.17	5.23–9.38	−0.121	91.11–95.99	32.16–45.94	1	3	2	3	1	0
*Phoebe bournei*	13	285–532	31.72–59.32	5.92–9.42	−0.081	88.25–105.1	26.34–48.62	0	2	5	6	0	0
*Cinnamomum camphora*	10	94–999	10.11–112.42	5.97–9.67	−0.117	83.09–104.1	34.33–44.73	0	1	5	1	0	3
*Populus trichocarpa*	19	376–1181	41.71–132.53	5.72–8.95	−0.057	85.37–102.5	28.78–46.43	2	5	1	11	0	0
*Eucalyptus grandis*	13	231–397	25.47–43.33	5.09–8.85	−0.092	83.94–100.3	36.47–49.07	0	1	0	12	0	0

PL, polypeptide length; MW, molecular weight; pI, theoretical isoelectric point; GRAVY, grand average of hydropathy; AI, aliphatic index; II, instability index; E.R., endoplasmic reticulum; chlo, chloroplast; plas, plasma membrane; cyto, cytoplasm; mito, mitochondria; nucl, nucleus.

**Table 2:** Synonymous and nonsynonymous substitution rates and ratios for PzCHSs.

Gene	Gene	Ka	Ks	Ka_Ks
*PzCHS2*	*PzCHS6*	0.962	1.838	0.523
*PzCHS1*	*PzCHS11*	0.105	1.756	0.060
*PzCHS3*	*PzCHS7*	0.211	1.114	0.190
*PzCHS3*	*PzCHS11*	0.056	1.004	0.055
*PzCHS7*	*PzCHS11*	0.230	1.313	0.175

## Data Availability

No new data were created or analyzed in this study. Data sharing is not applicable to this article.

## Supplementary Materials

The following supporting information can be downloaded at https://www.mdpi.com/article/10.3390/genes17091027/s1, Figure S1: Predicted three-dimensional structures of PzCHS proteins. Table S1: Physicochemical properties and predicted subcellular localization of CHS members. Table S2: Differential expression analysis of CHS members among different stem regions at different development stages. Table S3: Differential expression analysis of CHS members between sapwood and transition zone. Table S4: Differential expression analysis of CHS members between the CK and Dry treatments.
