# Supplementary material for: Genome-Wide Identification of the Chalcone Synthase Gene Family in Phoebe zhennan and Its Association with Golden-Thread Wood Color Formation and Drought Response
Source: Genes (Basel). 2026 Aug 28;17(9):1027. doi: 10.3390/genes17091027 (PMC13606206; doi:10.3390/genes17091027)
Supplement: Supplementary file 1 [file genes-17-01027-s001.zip › Supplementary Figure.pdf]

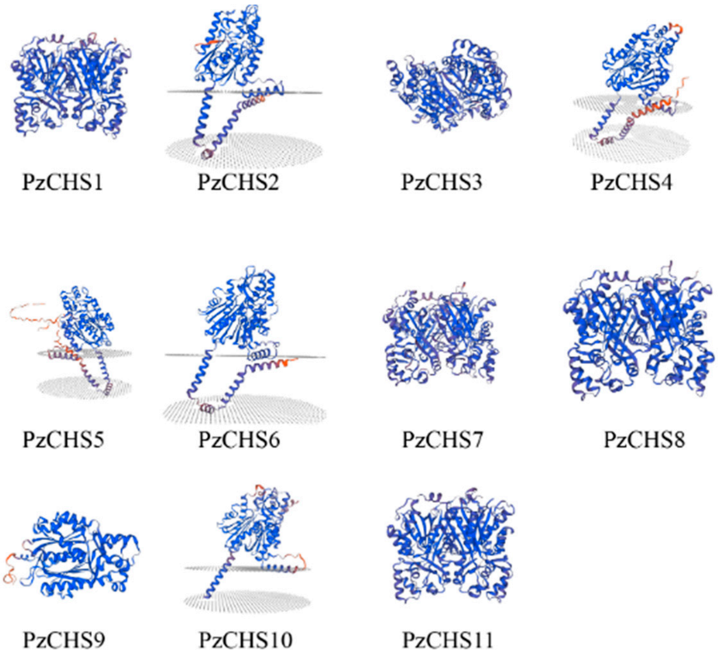

Figure S1. Predicted three-dimensional structures of PzCHS proteins
